# ‘I’m a bit of a champion for it actually’: qualitative insights into practitioner-supported self-collection cervical screening among early adopting Victorian practitioners in Australia

**DOI:** 10.1017/S1463423623000191

**Published:** 2023-04-27

**Authors:** Claire Zammit, Nicola Creagh, Claire Nightingale, Tracey McDermott, Marion Saville, Julia Brotherton, Margaret Kelaher

**Affiliations:** 1 Centre for Health Policy, Melbourne School of Population and Global Health, University of Melbourne, Level 4 207 Bouverie Street Carlton, Melbourne, VIC 3053, Australia; 2 Australian Centre for the Prevention of Cervical Cancer, 265 Faraday Street Carlton, Melbourne, VIC 3053, Australia (formally known as VCS Foundation)

**Keywords:** Cervical screening, cervical cancer, self-collection, self-sampling, qualitative, primary care, general practice, practitioners

## Abstract

**Background:**

Self-collection for cervical screening has been available in the Australian National Cervical Screening Program since 2017 and is now available to all people as an option for cervical screening through a practitioner-supported model. Documenting early adopting practitioner experiences with self-collection as a mechanism to engage people in cervical screening is crucial to informing its continuing roll-out and implementation in other health systems.

**Aim:**

This study aimed to describe the experiences of practitioners in Victoria, Australia, who used human papillomavirus (HPV)-based self-collection cervical screening during the first 17 months of its availability.

**Methods:**

Interviews (*n* = 18) with practitioners from Victoria, who offered self-collection to their patients between December 2017 and April 2019, analysed using template analysis.

**Findings:**

Practitioners were overwhelmingly supportive of self-collection cervical screening because it was acceptable to their patients and addressed patients’ barriers to screening. Practitioners perceived that knowledge and awareness of self-collection were variable among the primary care workforce, with some viewing self-collection to be inferior to clinician-collected screening. Practitioners championed self-collection at an individual level, with the extent of practice-level implementation depending on resourcing. Concerns regarding supporting the follow-up of self-collected HPV positive patients were noted. Other practical barriers included gaining timely, accurate screening histories from the National Cancer Screening Register to assess eligibility. Practitioners’ role surrounded facilitating the choice between screening tests through a patient-centred approach.

## Introduction

Australia is a global leader in the prevention of cervical cancer. The National Cervical Screening Program (NCSP), implemented since 1991, has halved cervical cancer-related cases and deaths (Australian Institute of Health and Welfare, 2019). In line with the World Health Organisation’s cervical cancer elimination strategy (World Health Organisation, 2020), Australia could become one of the first countries to eliminate cervical cancer as a public health problem (defined as an incidence of < 4 per 100 000 women) by 2035 (Hall *et al.,* 2019, Australian Institute of Health and Welfare, 2020). However, arresting and reversing Australia’s long-term gradual decline in screening participation are vital to Australia’s efforts to achieve elimination (Smith and Canfell, 2016, NHMRC Centre of Research Excellence in Cervical Cancer Control, 2021).

In response to evidence outlining the superiority of human papillomavirus (HPV) nucleic acid tests for cervical screening compared to cytology (Ronco *et al.,* 2014), in 2017 Australia transitioned from two-yearly Papanicolaou smears commencing at 18–20 years to five-yearly HPV DNA testing at 25 years. This change also allowed for the introduction of self-collection cervical screening where eligible people can collect their own vaginal sample for HPV test using a flocked swab. At the time of its initial introduction, self-collection was only available to people ≥ 30 years who were over-due for screening by ≥ 2 years or had never-screened and declined a clinician-collected test. A National Cancer Screening Register (NCSR) was also introduced. When using polymerase-chain reaction-based assays, self-collection has the same sensitivity for the detection of high-grade cervical lesions as a clinician-collected cervical sample (Arbyn *et al.,* 2018). As such, it is now available as a choice for all people who are due for cervical screening, hereafter referred to as universal access to self-collection (Department of Health, 2021).

Self-collection successfully increases cervical screening participation, particularly among under- or never-screened individuals, and has been trialed and implemented internationally through different models of care including mail-out models (both opt-in and opt-out) and door-to-door healthcare worker-facilitated models (Verdoodt *et al.,* 2015; Arbyn *et al.,* 2018; Yeh *et al.,* 2019). Most models have been shown to increase participation with the exception of opt-in mail-out models (Verdoodt *et al.,* 2015; Arbyn *et al.,* 2018; Yeh *et al.,* 2019), while mail-out models have been shown to increase participation, sub-optimal kit return and subsequent wastage, experienced in the Australian context (Sultana *et al.,* 2016), bring the cost effectiveness and overall impact of the model into question. The home-visit model has the advantages of individual engagement and potentially immediate collection and has mostly been implemented within low- and middle-income contexts combined with community-based education (Verdoodt *et al.,* 2015; Arbyn *et al.,* 2018; Yeh *et al.,* 2019). Self-collection is also an acceptable cervical screening modality for participants and providers due to its ability to overcome commonly experienced barriers to screening participation, promoting autonomy and empowerment, as well as fostering trusting relationships between patients and practitioners (Camara *et al.,* 2021).

In Australia, while the test itself is self-administered, it must be ordered by a doctor or nurse practitioner who is responsible for follow-up, hereby referred to as a practitioner-supported model (Australian Government, 2018). An Australian trial of practitioner-supported self-collection within primary care settings found that 85.7% of participants who were offered self-collection completed the test (Saville *et al.,* 2018). We previously reported the high levels of acceptability among both providers and practitioners and highlighted that the implementation of self-collection in primary care was limited by the restrictive eligiblity criteria, limited knowledge and awareness and confusion among practitioners regarding the clinical practice guidelines (Creagh *et al.,* 2021, Creagh *et al.,* 2022). Here, we present a more detailed analysis of the practitioner experience using self-collection. These findings are highly relevant for the continued use of self-collection within the Australian NCSP and provides learnings for other national programmes looking to prepare the primary care workforce for the introduction of a self-collection cervical screening option.

## Materials and methods

### Ethics approval

This study was approved by the University of Melbourne Medicine and Dentistry Human Ethics Sub Committee (19540446.2). All participants provided informed consent (written or verbal) prior to being interviewed.

### Study setting

The Australian Centre for the Prevention of Cervical Cancer (ACPCC) operates VCS Pathology. At the time of this study, VCS Pathology was the only laboratory in Australia accredited to process self-collected samples for cervical screening, meaning that the entire population of practitioners who had implemented self-collection was known to ACPCC. During this time, self-collection was only available to those aged 30 years ≤, who were overdue for cervical screening and refused a practitioner-collected test. Practitioners invited to participate were purposively sampled following an analysis of the state-wide distribution of referrals to testing for self-collected samples. Referring practitioners were stratified by practitioner type (general practitioner, nurse practitioner or other practitioner types, including obstetrician/gynaecologists and practitioners based in hospitals), location of practice (metropolitan or regional area) and the number of pathology requests for self-collection testing of patients (‘volume of use’: 1-5 requests, 6-10 requests, 11–15 requests). The number of women who had utilised self-collection within each health region per practitioner type was used to determine the proportion of practitioners selected from each region.

### Recruitment of participants

Using purposive sampling from the sampling framework, VCS Pathology obtained contact details of Victorian practitioners who had ordered self-collected screening tests between December 2017 and 30 April 2019. VCS Pathology initially sent letters to 50 practitioners, inviting them to participate in the study. Upon receipt of the letter, practitioners had 14 days to opt-out of future contact. If no reply was received during this period, practitioners’ contact details were provided to author CZ who contacted practitioners via phone or email to assess interest in participation and to arrange an interview time. A second round of 50 further invitations was sent which over sampled male practitioners to maximise their representation as study participants.

### Data collection and analysis

The author CZ conducted one-on-one semi-structured interviews with practitioners. Interviews were conducted in person or by telephone using an interview protocol (Appendix 1) covering themes including practitioner awareness and preparedness for self-collection and perceptions of self-collection as a mechanism to increase participation and implementation experience. The interview protocol was piloted prior to data collection. Interviews lasted between 26 and 70 minutes and were conducted between July and October 2019 with an average interview duration of 48 minutes. A cinema voucher valued at $84.00AUD was offered as an incentive to the participants.

All interviews were professionally transcribed and analysed using NVivo (QSR International Pty Ltd, Version 12) for thematic template analysis (Brooks *et al.,* 2015). Transcripts were cross-checked with the audio-recording as a means of correcting errors and familiarisation with the dataset. Themes outlined within the interview protocol and relevant literature were used to inform an *a priori* coding framework as a means of ensuring methodological and theoretical rigour. The coding framework was revised iteratively as emergent themes were identified with a finalised coding framework applied once no further themes were identified. Author MK cross-checked the formulated coding framework to ensure consistency across the sample of practitioners. A finalised coding framework was then applied to the remaining interviews (Appendix 2).

## Results

Ninety-one practitioners who did not opt-out were contacted and invited to participate. Of these, 18 practitioners (19.8%) consented to an interview. No data were collected on those who declined. Most practitioners interviewed were general practitioners (56%, *n* = 10) and were based in a metropolitan area (56%, *n* = 10). The most common practice settings reported were practitioners based in a community health (28%, *n* = 5) or private billing setting (28%, *n* = 5). Most practitioners, at the time of the interview, had requested between one and five self-collection samples for pathology testing (55.6%, *n* = 10). Table 1 summarises interviewee’ characteristics as previously reported (Creagh *et al.,* 2021).

**Table 1.** Demographics of cervical screening practitioners interviewed as reported in Creagh et al. 2021

Classification	Variable	Number (*n*, %) *n* = 18
Cervical screening provider	General practitioner	10 (56)
	Practice nurse, nurse practitioner, midwife	8 (44)
Location	Metropolitan	10 (56)
	Regional	8 (44)
Primary care setting (self-reported^||^)	Gender diverse health^¶^	2(11)
	ACCHO^*^	3 (17)
	Community health^§^	5 (28)
	Private billing practice^‡^	5 (28)
	Public billing practice^†^	3 (17)
Volume of use (number of pathology request sent for self-collected samples received by VCS for testing per practitioner)	1–5	10 (55.6)
	6–10	6 (33.3)
	11–15	2 (11)

* Aboriginal Community Controlled Health Organisation specialising in the health needs of a specific Aboriginal or Torres Strait Islander community.

† Government funded and subsidised primary healthcare service.

‡ Privately or corporately owned and funded primary care health service.

§ Primary care health service that caters to the health needs of a specific community or region.

|| Practitioners were asked ‘what type of practice setting do you work in?’

¶ Primary care health service catering to the health needs of the trans, gender diverse and non-binary community.

### Practitioners’ perceptions of the self-collection cervical screening pathway within the renewed NCSP

As early adopters of the pathway, practitioners were overwhelmingly supportive and enthusiastic about the introduction of self-collection for cervical screening. They felt self-collection addressed a multitude of barriers experienced by under- or never-screened participants.

*‘I was very excited to hear about self-collection because I do have a number of patients who, for social and cultural reasons, have not been screened in the past and have been very resistant to screening, so I was very excited about being able to offer them self-collection’*. (Practitioner 4)

Practitioners’ support for self-collection was influenced by the fact that their patients perceived self-collection to be an acceptable alternative cervical screening modality to the clinician-collected test. This was also true for practitioners who worked with specific priority populations, including Aboriginal and Torres Strait Islander and gender diverse participants.

*‘Majority of the women found it quite easy to do the self-collection. A lot of women found that it did not make them feel shamed, which is a phenomenon with Aboriginal and Torres Strait Islander women. They would talk about having past experiences, sexual abuse and that they then felt empowered’* (Practitioner 11)

### Practitioners’ knowledge and awareness about the availability of self-collection cervical screening

While the majority of the practitioners reported a high level of knowledge and awareness of the NCSP, and by extension the self-collection pathway, practitioners believed there was a large variation in knowledge and awareness in the primary care workforce regarding self-collection overall.

*‘A lot of our GPs haven’t heard about it before, so I might need to hand them the resource about GPs and self-collection… We had a lot of relieving GPs at the moment, so they come from all over Australia and they’re unaware of the self-collection’* (Practitioner 14)

There was a persistent belief among practitioners that self-collection was an inferior test. This meant that practitioners viewed self-collection as being a ‘*lesser alternative’* to a clinician-collected test and considered it acceptable that the eligibility for self-collection was restricted to under-screened populations.

*‘it’s [self-collection] a less than optimal test so you don’t want to use it more than you need to’* (Practitioner 3)

### The role of a practice champion and a ‘whole of practice’ approach to the implementation of self-collection cervical screening in primary care

Most practitioners saw themselves, and their strong motivation for engaging under-screened participants, as key drivers in their decision to implement self-collection within their clinical practice. As early adopters of self-collection, practitioners noted that they were pivotal in championing the implementation of self-collection and provided education to colleagues within their practice setting to promote the adoption and uptake of self-collection.

*‘I organised the staff response…I did a little cheat sheet and had a meeting with the reception staff, so they were able to understand it. They now are relaying results and questions at the front desk. I still got it… that little information sheet, to help them explain what was happening’* (Practitioner 12)

The extent to which self-collection was implemented at a ‘whole of practice’ level’ was, however, contingent upon the practices’ available resourcing. Some practitioners reflected on their ability to harness existing practice structures and focus on cervical screening to promote self-collection to extend its reach. For example, some practitioners utilised text message recalls to enhance self-collection awareness among patients. In circumstances where there was minimal resourcing to apply a ‘whole-of-practice’ approach to preventative health care, practitioners were limited in their capacity to provide self-collection and did so at the individual level.

*‘Lots of people change address, not many people change their mobile phone even though they had changed address… We’ve added those women, the ones who were notified from through VCS, we added them onto the text message cycle. They get a text message to say, ‘Hi, it’s been longer than four years. You may be eligible for self-collect’* (Practitioner 9)

### Practitioners’ perception on their role in supporting screening participants decision-making about self-collection cervical screening

Practitioners saw their role as providing eligible participants with a choice between a clinician-collected or self-collected test, noting that the availability of self-collection centred around *‘participant’s choice’*. Practitioners noted that screening participants’ unwillingness to engage in cervical screening overall influenced the practitioner’s decision whether to offer self-collection as an alternative screening modality.

*Interviewer: What are the barriers…in bringing up cervical screening with under- or never-screened women?*

*Practitioner: Their [the participant’s] reluctance [to engage in screening], but that doesn’t stop you having a conversation. Just means you’re very respectful with the conversation you have, and…you respond to their experience. You don’t just keep going on your track.* (Practitioner 12)

Practitioners’ ability to facilitate a trusting environment, where screening participants felt safe and supported, was considered key in being able to have discussions surrounding the implications of self-collection should a positive result be discovered. Practitioners deemed this to be critical in assisting screening participants decision-making capacity as well as their own decision to offer self-collection:

*And then most of our engagement with women to have their screen… it’s done by time because it allows a clinical conversation to go on…It’s part of building a trusting relationship because often I’m bringing [in] women who just enrolled in the clinic and saying, you filled in the form and you gave us the okay to get your history, we’ve done that and we see that you’ve actually got some things that need following through, and so you start that process.* (Practitioner 11)

### Concerns and barriers raised by early adopting practitioners

While practitioners portrayed a willingness to continue to provide self-collection, there was concern regarding the management processes for HPV positive patients to minimise loss to follow-up, especially for those who require an additional speculum-collected test after an HPV (non-16/18) test result.

*‘In terms of the self-taken, people have the most difficulty with if it comes back with HPV detected, then they’ll have to have a conventional examination. And I still have one patient who has non16/18 HPV and who has declined to do that and this person is actually a social worker and let’s say somebody who is educated who knew right from the start that this would be the procedure and still has said, ‘I think I’ll take my chances’*. (Practitioner 10)

Given the self-collection policy at the time of data collection restricted eligibility to under-screened participants, practitioners considered being able to access screening histories from the NCSR to identify eligible participants as essential in offering self-collection. However, practitioners noted the length of time taken and administrative burden when trying to access participants’ cervical screening history. This limited practitioners’ capacity to offer self-collection opportunistically, which led to practitioners describing the NCSR as a *‘big hindrance’* in being able to offer self-collection more readily.

*‘I’ve had some issues, contacting them, with the amount of time that it takes me to actually speak to a human, with the amount of information that the person on the other end of the phone is able to get me in a timely manner. I think it’s been a lot slower than the Victorian cervical screening registry that I used to find. That’s been difficult’*. (Practitioner 4)

## Discussion

This is the first detailed insight into early adopting practitioners’ perceptions and experiences of the self-collection cervical screening pathway within a national screening programme. We report that early adopting practitioners were enthusiastic about self-collection, driven by its high acceptability to screening participants who were previously hard to engage via clinician-collected cervical screening. Practitioners viewed their role as providing participants with information and support to choose the cervical screening modality that best suited their needs and circumstances, with a trustful environment considered key in supporting participants’ decision making surrounding cervical screening. While practitioners reported a high level of knowledge and understanding about self-collection, the misconception remained that self-collection had inferior sensitivity to the clinician-collected screening test. Practitioners also perceived a large variation in understanding about self-collection among the general primary care workforce. Interviewed practitioners saw themselves as the champions of self-collection within their practice setting; however, practitioners’ ability to implement self-collection at a ‘whole of practice’ level was dependent upon available resourcing within the practice. Practitioners held concerns about the potential for increased loss to follow-up after an HPV positive result from a self-collection test, especially for those who require a secondary clinician-collected cervical screening test and reported an added administrative burden in obtaining cervical screening histories from the NCSR. This added burden reduced practitioner’s ability to offer self-collection opportunistically which has been previously described (Smith *et al.,* 2019; Dodd *et al.,* 2020; Obermair *et al.,* 2021).

We described our study participants as early adopters of self-collection, because the uptake of self-collection under the renewed NCSP has been very low (Smith *et al.,* 2020). At the time of the study, approximately 18 months after the introduction of self-collection within the Australian NCSP, only 1067 self-collection test had been performed in the state of Victoria, Australia (Smith *et al.,* 2020). In the first two years, less than 1% of women eligible performed or accessed self-collection nationally even though estimates indicated over 1 million screening participants were eligible for self-collection nationally (Smith *et al.,* 2020). While participants within this study reported a high level of acceptability of self-collection, other research with practitioners based in rural NSW with limited experience of the pathway reported that they viewed self-collection as an appropriate second option but preferred the clinician-collected screening modality, citing concerns surrounding the sensitivity of self-collection and inadequate sample collection by patients (Foo *et al.,* 2021). Furthermore, a survey conducted by Obermair *et al.* (2021) found that only 65% of practitioners believed that self-collection was a suitable screening modality for under-screened populations with a previous survey performed by Sultana *et al.* (2020) finding only 57.4% of practitioners’ post-renewal were confident in recommending self-collection to under-screened women. For self-collection to effectively address issues of persistent inequity that exist in participation for specific populations, including Aboriginal and Torres Strait Islander peoples (Whop *et al.,* 2016), gender and sexually diverse people (Kerr *et al.,* 2022), people who live in socio-economically disadvantaged or rural and remote areas (Australian Institute of Health and Welfare, 2020) within the programme, as intended, there must be wider-reaching effort to increase the number of practitioners offering self-collection as part of routine care.

Providing widely accessible education that specifically addresses misconceptions and barriers that have been identified in this study and others may help to achieve greater adoption of self-collection cervical screening by primary care. While most of the participants within our study, as early adopters, reported a high level of knowledge of self-collection, they did perceive that the broader primary care workforce did not hold the same level of knowledge and awareness of the pathway, which may be impacting broader adoption of self-collection. Like other literature (Foo *et al.,* 2021; Jaenke *et al.,* 2021), our study highlighted how some practitioners incorrectly believed that self-collection samples were less sensitive that clinician-collected samples, despite strong evidence of equivalent sensitivity (Arbyn *et al.,* 2018). While there was education to support the rNCSP and self-collection, our work and that of others (Obermair *et al.,* 2021; Dodd *et al.,* 2020) demonstrate confusion remained about how to implement the guidelines. With the change in policy allowing universal access to self-collection, there has been further clarification on guideline implementation allowing flexibility in how self-collection can be facilitated for screening participants. New education modules have since been developed (GPEx One, 2022), and a number of Federal resources are available to practitioners such as the NCSP toolkit (Australian Government, 2022). Ongoing evaluation of these will be important to ensure they are meeting the needs of the workforce. It is of the utmost importance that practitioners’ access, trust and understand the latest science behind the policy decision to provide self-collection within the NCSP. This is consistent with recommendations from previous studies surrounding updated and targeted education for self-collection is provided to cervical screening practitioners (Obermair *et al.,* 2021) and will ensure wider reaching access to self-collection along with meaningful improvements in screening participation. Within the Australian programme context, updated education of practitioners regarding the expansion of self-collection, alongside the integration of the NCSR into the major types of practising medical software to increase access to screening histories, will better equip primary care ahead of self-collection cervical screening now being a universal choice for all people undergoing cervical screening. Similar actions should be taken within contexts where practitioner-supported self-collection is favoured.

We also hypothesise that universal access to self-collection is likely to increase participant demand for self-collection, highlighting the importance of ensuring that primary care is equipped and resourced to provide self-collection cervical screening as part of their services with the policy change simplifying the implementation of self-collection. Additional enablers to the adoption of self-collection are likely to include updated education and training of primary care and expanded capacity of other pathology providers to process self-collected tests. Moreover, community-based campaigns, raising awareness about self-collection and driving consumer demand may also have a positive impact on the number of practitioners offering self-collection. Practitioners also demonstrated concerns about ensuring people who test HPV positive through self-collection are supported through cytology and ongoing follow-up that is required. In Australia, should an HPV positive result eventuate from a self-collected sample, participants are required to undergo a clinician-collected sample for cytology (Canfell *et al.,* 2021). Evidenced within an Australian pilot study, high levels of completion of follow-up were achieved for people who participate through self-collection (Saville *et al.,* 2018). Although as lower levels of acceptability of self-collection have been noted among HPV positive women who required clinician-collected sample for cytology (Creagh *et al.,* 2021), ongoing monitoring of patient experience and further educational support for practitioners are warranted.

Another important finding from this study was that, while practitioners considered themselves as champions of self-collection, their ability to adopt a *‘*whole of practice’ approach to the implementation of the restricted self-collection cervical screening pathway remained resource dependent. Both practice champions and utilising a ‘whole of practice’ approach have been found to been characteristics of successful interventions in improving cancer screening participation within primary practice (Shaw *et al.,* 2013; Hills *et al.,* 2015; Weiner *et al.,* 2017; Bakhai *et al.,* 2018). This point is pertinent as considerations are given to how the Australia health system can foster greater adoption of self-collection cervical screening by primary care. We recommend this is a point for further investigation among local and international primary care policy makers, clinicians and researchers.

### Strengths, limitations and policy implications

Australia is one of 11 countries that offers self-collection as a choice for routine cervical screening with the majority of these being within low-to-middle income settings (Serrano *et al.,* 2022). There is limited research or evidence regarding the experience of implementing self-collection cervical screening as a part of an organised national programme. The findings of this research have direct implications for the new universal access policy in Australia and fill a prominent gap within the literature. These learnings and recommendations can be applied to other countries who are looking to transition to primary HPV-based screening programmes with a self-collection pathway. New Zealand has announced the transition to HPV-based screening in 2023 including the introduction of practitioner-supported self-collection (National Screening Unit – Ministry of Health, 2021). Although our study only included practitioners from the state of Victoria, these results provide significant insight into implementation barriers and facilitators for policy makers and service providers looking to apply self-collection more broadly. It is also important to consider that these implementation barriers may still exist; therefore, further research into how primary care is tracking with the new policy change in Australia is warranted. Additionally, our sample size was limited by the fact that few Victorian practitioners had used self-collection at the time of the study and hence we recruited a low number of practitioners (18 out of 100 practitioners invited) to the study despite consistent themes across interviews.

## Conclusion

This study elucidates the experience of early adopting practitioners who utilised the self-collection pathway within the Australian NCSP. Given the rapidly emerging body of evidence demonstrating self-collection is effective in increasing cervical screening participation, this study highlights opportunities to maximise the uptake of self-collection in primary care, where practitioners are critical in driving the reach of self-collection. Practitioners and practices need to be supported and equipped with appropriate and accurate information to inform their practice and support screening participants. Doing so will ultimately maximise the potential for the self-collection pathway to increase screening participation and progress Australia’s efforts towards cervical cancer elimination.

## Supplementary material

For supplementary material accompanying this paper visit http://doi.org/10.1017/S1463423623000191. click here to view supplementary material
