# Supplementary material for: ‘I’m a bit of a champion for it actually’: qualitative insights into practitioner-supported self-collection cervical screening among early adopting Victorian practitioners in Australia
Source: Prim Health Care Res Dev. 2023 Apr 27;24:e31. doi: 10.1017/S1463423623000191 (PMC10156465; doi:10.1017/S1463423623000191)
Supplement: Supplementary file 1 [file S1463423623000191sup001.docx]

**Appendix 1. Interview Protocol Version 3**

*Practitioners* - (General practitioners/ nurses/ gynaecologist and obstetrician/hospital staff who have participated in the self-collection pathway between 1^st^ December 2017 and date of data extraction)

Practitioners that are participating in the study will be given the opportunity to recall and describe their experience of the self-collection cervical screening pathway from their perspective. As such, the flow of the interview will be largely dictated by the participant. The interviewer will use the following prompts to guide the interview if necessary, ideally in an order that follows the chronological order of the self-collection pathway.

*The interviewer will begin by discussing the purpose of the research study, talking the participant through the Plain Language Statement and ensuring that they have given informed consent to participate. They will then gain verbal consent and ask whether the participant consents to being audio recorded.*

**Theme 1: Introduction and demographic information**

- Thank you <name> for taking part in the research.
- What is your current involvement in providing cervical screening and self-collection?
  - Probe: what is your role as a practitioner?
  - Probe: What type of practice setting do you work in?

**Theme 2: Practitioners understanding of the self-collection cervical screening pathway**

- When and how did you hear about the renewal of the National Cervical Screening Program and in particular, the self-collection screening pathway
  - Probe: What was your initial opinion about the renewal and the self-collection screening pathway 🡪 Has your initial opinion changed for the rNCSP and the self-collection pathway. Why/why not?
- Was there any information provided to you or that you/your organisation sought out to assist in the implementation of the rNCSP and in particular self-collection pathway?

**Theme 3 - Practitioner and/or preparation to the implementation of the self-collection screening pathway**

- How did you/your organisation prepare for the rNCSP and the self-collection pathway?
  - Probe: Were there any processes and/or activities performed by yourself and/or your organisation to assist in the implementation of the self-collection screening pathway?
- In your experience, what are or have been the challenges or barriers encountered in the early stages of the implementation of the self-collection screening pathway in your practice/organisation?
- Probe: Did you experience any difficultly accessing information or resources in order to implement the self-collection cervical screening pathway, any difficulties setting up processes or activities in order to identify participants who may be eligible for the pathway?
- In your experience, what are or have been the facilitators that have assisted in the initial implementation of the self-collection screening pathway in your practice/organisation?
  - Probe: was there processes already put in place that assisted you and/or your organisation to identify participants, was resources and equipment easily accessible etc.

**Theme 4 - Processes adopted by practitioner and/or primary care practice to identify patient who is under-screened or never-screened**

- What is your and/or your organisations approach to the National Cervical Screening Program
  - Probe: Do you have capacity in your clinical practice to focus on preventative health, and as such focus on cervical screening? Why/why not? – Barriers and enablers to allow a focus on the NCSP
- What, if any, have been the main changes in your practice that have occurred due to the introduction of the renewed National Cervical Screening Program?
  - Probe: Have there been any challenges since the introduction of the renewed National Cervical Screening Program?
- Who in, and how does, your practice identify a woman who is under-screened or never-screened for cervical screening?
  - Probe: If your identification of women who are under-screened or never-screened opportunistic or systematic? 🡪 Which approach do you prefer?
  - Probe: What are the challenges that you/your organisation incurs when identifying women who are under-screened or never screened for cervical screening 🡪 What are the facilitators that assist you/your organisation in identifying women who are under-screened or never-screened for cervical screening
- Probe: In your opinion, what would be the most ideal approach to identify under-screened or never-screened women that may be eligible for self-collection?
- (If this approach is not the current approach adopted by the practitioner/practice in identifying women who are under-screened or never-screened for cervical screening). Probe - What restricts you/your organisation from implementing the most ideal approach to identify women who are under-screened or never-screened for cervical screening?
- In your experience, is there any women/groups/sub-groups of women who come to you/you practice that are more frequently under-screened or never-screened women 🡪 Yes – does this alter you approach

**Theme 5 - Processes adopted by practitioner to offer self-collection cervical screening to patient who are under-screened or never-screened**

- What has been your experience of offering women who are under-screened or never-screened, cervical screening
  - Probe: Barriers or facilitators in bringing up cervical screening with under-screened or never-screened women?
  - Probe: How do you deal with the requirement that women must refuse a practitioner collected sample in order to be eligible for self-collection? 🡪 Does this alter your approach?
- What is the process required for your patients to perform the self-collection test?
  - Probe: Where do your patients take the sample at your practice? 🡪Is there any logistical or privacy issues that have arisen? 🡪 Why/why not?
  - Probe: Have any patients had any difficulties taking the self-collected sample? 🡪 How do your respond to these difficulties?
- Have you encountered any women who you have offered self-collection screening to that have refused self-collection screening?
  - (Answer = Yes) Probe: What have been the reasons that women have refused the self-collection screening test?
- Have you had women who are concerned about the accuracy of the self-collection cervical screening test?
  - Probe: Is this part of your discussion with your patient’s when offering the patient, the self-collection screening test?
- Probe: What is your understanding of the accuracy of the self-collection cervical screening test compared with the clinician collected cervical screening test?

**Theme 6 - Processes adopted by practitioner and/or primary care practice to send self-collection HPV sample to pathology**

- After a woman has completed the self-collection cervical screening test, what is your/your practice’s approach to having the sample sent off to pathology?
- What, if any, what have the barriers and/or facilitators been to have the sample sent off to pathology?

**Theme 7 - Processes adopted by practitioner/primary care practice to discuss the screening result with the patient**

- What is your approach to delivering the results to patients who have utilised the self-collection cervical screening pathway?
  - Probe: Is this the approach that you to take to deliver the resulted to patients who have participated in cervical screening not by self-collection?
  - Probe: Does your approach differ for women who utilise the self-collection cervical screening pathway who either test positive for HPV or when the HPV testing was unable to be performed? 🡪 Why?
- What is your experience discussing and interpreting the screening result with your patients who have utilised the self-collection cervical screening pathway and how do you communicate and agree on the next steps?

**Theme 8 - Availability, affordability and accessibility to colposcopy specialists**

- For your patients who have utilised self-collection, have any patients required any further testing or follow-up medical treatment?
  - If yes – what was your experience of <inset event> - Either obtain clinician-collected sample for liquid based cytology or referral to colposcopy assessment for care? 🡪 Any barriers and/or facilitators to <insert event>, was this process accepted and suitable for your patient/s?
  - If no – What is your opinion or perception of how challenging it might be to either obtain a clinician-collected sample for liquid based cytology or referral to colposcopy assessment for care?
  - Probe: are there colposcopy specialists accessible/available/affordable for patients who utilise the self-collection cervical screening pathway?

**Theme 9 - Overall**

- Who or what has helped the implementation of the self-collection screening pathway in your organisation?
- Who or what had hindered the implementation of the self-collection screening pathway within your organisation?
  - Probe: have there been any unforeseen issues with implementing the self-collection cervical screening pathway?
- Is there anything else you would like to share about your experience utilizing the self-collection screening pathway that we haven’t talked about?

**Appendix 2. Finalized Coding Framework**

| **Theme** | **Nodes** |
| --- | --- |
| 1. Practitioner’s perception of under-screened and never-screened screening participants | - Demographics of under-screened women - Perceived barriers to cervical screening from practitioner’s perspective |
| 2. Practitioner’s views and perception of self-collection and rNSCP | - Motivation - Up-skilling and knowledge sharing - Acceptability and effectiveness - Cultural sensitivity - Effectiveness (reach and accuracy) - Acceptability (uptake) |
| 3. Integration of the self-collection pathway into clinical practice | - Roles and responsibilities - Practitioner behaviour - Organisational procedures & Capacity - Identification - Refusal of clinician collected cervical screening test - Offer of self-collection - Self-collection process - Discussion of results - Follow up or referral |
| 4. Experienced barriers and facilitators to implementation of self-collection | - Individual level - Organisational level - System level |
